# Standardised Resting Time Prior to Blood Sampling and Diurnal Variation Associated with Risk of Patient Misclassification: Results from Selected Biochemical Components

**DOI:** 10.1371/journal.pone.0140475

**Published:** 2015-10-13

**Authors:** Ida B. Andersen, Claus L. Brasen, Henry Christensen, Lene Noehr-Jensen, Dorthe E. Nielsen, Ivan Brandslund, Jonna S. Madsen

**Affiliations:** 1 Department of Clinical Immunology and Biochemistry, Lillebaelt Hospital, Vejle, Denmark; 2 Department of Clinical Immunology and Biochemistry, Lillebaelt Hospital, Kolding, Denmark; 3 Institute of Regional Health Research, University of Southern Denmark, Odense, Denmark; Osaka University Graduate School of Medicine, JAPAN

## Abstract

**Background:**

According to current recommendations, blood samples should be taken in the morning after 15 minutes’ resting time. Some components exhibit diurnal variation and in response to pressures to expand opening hours and reduce waiting time, the aims of this study were to investigate the impact of resting time prior to blood sampling and diurnal variation on biochemical components, including albumin, thyrotropin (TSH), total calcium and sodium in plasma.

**Methods:**

All patients referred to an outpatient clinic for blood sampling were included in the period Nov 2011 until June 2014 (opening hours: 7am–3pm). Each patient’s arrival time and time of blood sampling were registered. The impact of resting time and the time of day for all components was analysed using simple linear regression. The “maximum allowable bias” was used as quality indicator for the change in reference interval.

**Results:**

Significant diurnal variation was found for albumin (n = 15,544; p<2×10^−16^), TSH (n = 20,019; p<2×10^−16^), calcium (n = 13,588; p = 2.8×10^−12^) and sodium (n = 51,917; p<2×10^−16^). Further significant influence for resting time was found for albumin (p = 2.6×10^−4^), TSH (p = 0.004), calcium (p = 8.9×10^−7^) and sodium (p = 8.7×10^−16^). Only TSH and albumin were clinically significantly influenced by diurnal variation. Resting time had no clinically significant effect.

**Conclusions:**

We found no need for resting 15 minutes prior to blood sampling. However, diurnal variation was found to have a significant and considerable impact on TSH and, to a minor degree, albumin. This has to be taken into account to ensure that reference intervals provided by the laboratory are valid on a 24-hour basis.

## Introduction

During the last decade, preanalytical quality in laboratory medicine has been subject to increased attention. Before this time, the analytical phase was the main point of focus but it has become clear that the pre- and postanalytical phases are much more vulnerable than previously expected. This resulted in the formation of several working groups (WG) which focus on the preanalytical phase. Among these groups are the European Federation of Clinical Chemistry and Laboratory Medicine (EFLM) WG; “Preanalytical Phase” and the International Federation of Clinical Chemistry and Laboratory Medicine WG; “Laboratory errors and patient safety” [1]. The “Preanalytical Phase” suggests a European harmonisation on patient preparation prior to morning sampling [2].

The core service of any clinical chemical laboratory is blood sampling and analysis. In connection with this, reference intervals or decision limits need to be established. Previous studies have shown that some biochemical components exhibit diurnal variation [3,4] and/or are sensitive to postural change. The latter is due to the effect of gravitational force and hydrostatic pressure on the plasma volume [5]. Therefore laboratories are recommended to apply the rules of standardisation based on preanalytical factors, which state that blood samples should be taken in the morning after at least 15 minutes of rest [6]. However, in relation to outpatient laboratory blood sampling, patients and hospital administrators have expressed a desire to minimise waiting time prior to blood sampling and to extend clinic opening hours. Therefore the evidence base for recommending a standardised period of rest prior to blood sampling has been challenged. The primary aim of this study was therefore to investigate the impact of resting time prior to blood sampling. To do this, we focused on a few common biochemical components representing different aspects of reaching steady state: Albumin (large molecules), thyrotropin (TSH) (hormone subject to pulsatile release), sodium and total calcium (small molecules with active transport systems). Furthermore, the impact of diurnal variation and therefore the importance of the time of day for blood sampling were investigated.

## Materials and Methods

Consecutive patients referred to an outpatient clinic for blood sampling were included in the period from Nov 2011 to June 2014 (clinic’s opening hours: 7am-3pm). Waiting time for each patient was calculated by registering time of arrival and time of blood sampling using the Q-MATIC Suite (Q-MATIC, Skovlunde, Denmark). Patients under the age of 20 (TSH) or 15 years (albumin) were excluded from the data analysis due to age-specific differences in reference intervals. Moreover, patients arriving for ECG-measurement were excluded. Only patients who waited from 0–60 minutes were included. The resting time was estimated to be the same as the waiting time, and this period is referred to as “resting time” throughout the article.

Diurnal variation and the effect of resting time were investigated for the following components: TSH, albumin, total calcium and sodium.

In line with the recommendations, the reference conditions for blood sampling were set at 7-9am after a resting time of 15–30 minutes [2,5,6].

Statistical analyses were performed using the statistical software “R” version 3.1.1. The significance level was set at p = 0.05.

For all TSH-calculations, except when patients are classified in relation to the reference interval, patients with TSH-results <0.03 and >100 mIU/L were excluded from the data analysis, since we had no precise value for these. The underlying assumptions regarding linearity were assessed by qq-plots and residual plots, and when the qq-plot indicated deviation from the normality of the residuals, a power transformation of data was made. The linear regression was then estimated on the transformed scale and the new model was once again checked for goodness of fit by residual plot.

Calculated values for albumin, calcium and sodium were estimated using linear regression taking into account the time of day (and for albumin also age). In addition, for albumin, only patients waiting 0–10 minutes were included in the linear regression, since the entire change occurred within the first 10 minutes.

To investigate the diurnal variation, the mean variation for each component was calculated at one-hour intervals. We investigated whether, during the day, the mean changed more than 1/3×SD_pop_ from the mean under the reference conditions. For the reference interval to be valid, the mean change must be ≤1/3×SD_pop._ This is the maximum allowable bias (MAB) for use as a common reference interval value [6,7,8].

Gowans *et al*. presented the idea about the use of the same population-based reference intervals in several laboratories in a homogeneous population area [7,8]. From this we developed the formula ≤1/3×SD_pop_ from the linkage to Gowans’ principle and the fact that the reference interval has a span of 4SD_pop_ [6]. From Gowans *et*. *al*., the principle is given that 0.250SD is the “desirable” ratio and 0.375SD is the “minimum” ratio for accepting the *number of individuals outside conventional population-based reference limits*. Therefore we chose 1/3SD, an intermediate value, as the ratio. To find the SD_pop_, we divided the span of the reference interval by 4.

Ex. calculation of MAB for albumin (we applied a compromise between the three reference intervals listed in Table 1):

$$\text{Reference interval}:\;35-49\;g/L⟹{4SD}_{pop}=14⟹MAB=1/3\times {SD}_{pop}=1.17$$

**Table 1:** Different data for the biochemical compounds.

	No. of patients	Reference interval	MAB (≤1/3×SD_pop_)
**TSH**	19,180	20+ years, 0.30–4.0 mIU/L	0.31
**Calcium**	13,588	2,15–2,51 mmol/L	0.03
**Sodium**	51,917	137–145 mmol/L	0.67
**Albumin**	42,399/15,544*	15–40 years, 36–50 g/L	1.17**
	-	40–70 years, 36–48 g/L	-
	-	70+ years, 34–45 g/L	-

* 0–10 minutes resting time (both numbers includes all patients above the age of 15)

** calculated from a compromise between the three reference intervals

The maximum allowable bias (MAB) indicates the largest difference from the reference interval that is allowed. If the difference exceeds this value, the reference interval is no longer valid. For albumin only values from patients who waited 0–10 minutes (n = 15,544) are used for the linear regression, since the entire change occurred within this time interval.

If the change in mean diurnal variation or resting time is larger than the MAB, this indicates that the change is of clinical importance.

For TSH a “reference interval” classification of the patients was performed to investigate whether the distribution of patients changed during the day. The patients were again grouped at one-hour intervals and then subdivided into three groups: “below” (below 0.3 mIU/L), “within reference interval” and “above” (above 4.0 mIU/L).

To estimate and evaluate the effect of resting time prior to blood sampling and diurnal variation, the percentage change of the mean values relative to the mean under reference conditions (7-9am, 15–30 minutes) was calculated. This was performed for four resting time intervals (0–5 minutes, 5–15 minutes, 15–30 minutes and 30–60 minutes) at two-hour intervals during the opening hours (7–9am, 9–11am, 11am–1pm and 1–3pm).

All data used in this study were analysed anonymously and according to the health research ethics committee system in Denmark and the Regional Scientific Ethical Committees for Southern Denmark the project did not need approval by the committees.

## Results

A total of 42,399 samples for albumin, 13,588 for total calcium and 51,917 for sodium were included in the study. In addition, 20,019 TSH samples were included for the “Classification” relative to the reference interval, whereas, for the rest of the TSH-calculations, TSH-results <0.03 mIU/L (n = 826) and >100 mIU/L (n = 13) were excluded, leaving a total of 19,180 samples for these calculations.

The qq-plots and residual plots indicated deviation from the normality of the residuals for TSH but not albumin, calcium and sodium.

A Box-Cox analysis suggested a power transformation factor of 1/9.9 to normalise the TSH residuals. When the new model for TSH was again checked for goodness of fit by residual plot, there was no indication that further normalization was necessary.

Using linear regression, we found that plasma values for albumin, TSH, total calcium and sodium all showed significant diurnal variation (p<2×10^−16^ for albumin (n = 15,544, since the calculation is based solely on patients waiting 0–10 minutes), p<2×10^−16^ for TSH, p = 2.8×10^−12^ for calcium and p<2×10^−16^ for sodium).

**Fig 1:**
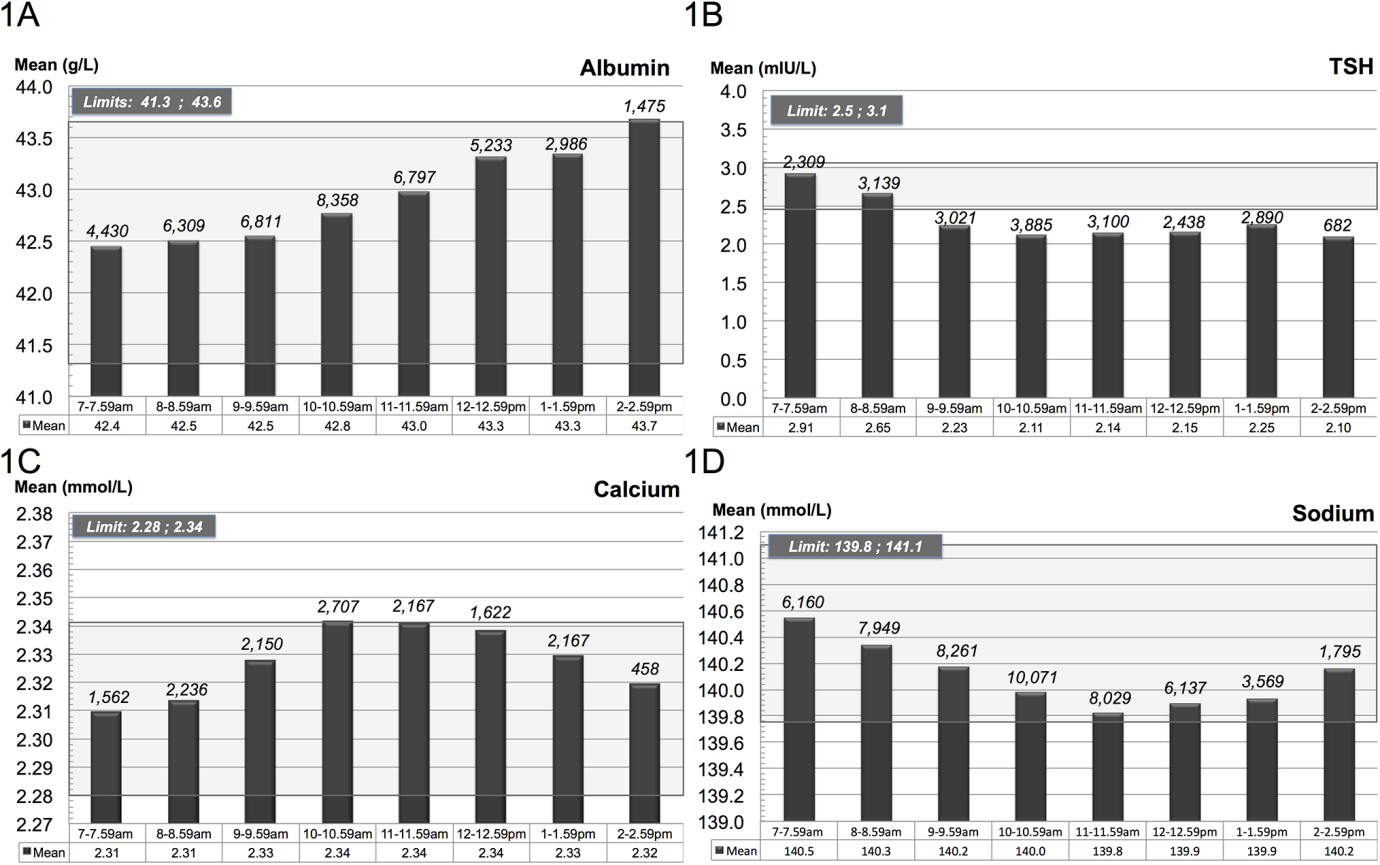
Diurnal variation. The figures illustrate the diurnal variation of A) albumin, B) thyrotropin, C) calcium and D) sodium divided into one-hour intervals. The values above the columns show the number of patients in each time interval. The shadowed background field area indicates the upper and lower limit defined by the maximum allowable bias.

The mean value of albumin showed an almost linear increase during the day from a mean value of 42.5 g/L (at 7–9am, n = 10,739) to 43.5 g/L (at 1–3pm, n = 4,461) (Fig 1A).

For TSH the mean value decreased from 2.76 mIU/L (at 7–9am, n = 5,448) to 2.22 mIU/L (at 1–3pm, n = 3,572) (Fig 1B). The observed decline was most pronounced between 7am and 9am whereupon more stable values were recorded. However, after 9am, all mean values were outside the lower MAB limit i.e. the differences from the reference value were greater than the allowable bias (Fig 1B).

For calcium, the mean value increased from 2.31 mmol/L (at 7–9am, n = 3,781) to 2.33 mmol/L (at 1–3pm, n = 1,321) (Fig 1C). For sodium, the mean decreased from 140.4 mmol/L (at 7–9am, n = 14,109) to 140.0 mmol/L (at 1–3pm, n = 5,364) (Fig 1D).

For albumin, sodium and calcium the variation in mean is small (<3%) whereas for TSH the mean varies nearly 28% depending on the time of day (Fig 1).

**Fig 2:**
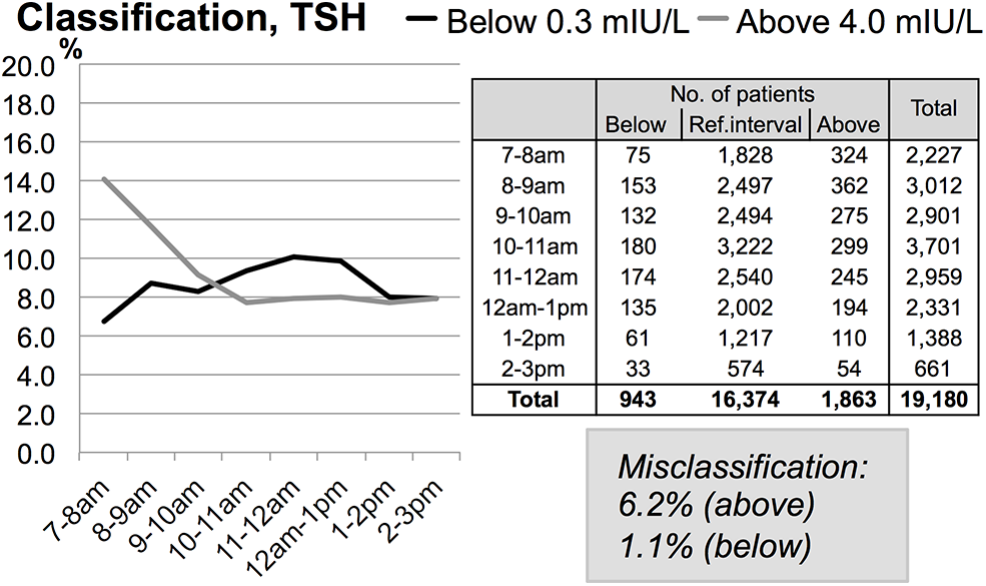
Misclassification of patients relative to the reference interval. Dividing the patients in three subgroups based on the reference interval produces “Below”, “Ref. interval” and “Above”. The table indicates the number of patients in each subgroup. The graph shows the same numbers in % for the subgroups “Below” and “Above”. The values in the “Misclassification” box are the number of patients misclassified at 2–3pm relative to 7–8am.

Fig 2 illustrates how the TSH-samples are classified in relation to the reference interval at one-hour intervals. When comparing values obtained during the morning to values obtained in the afternoon, a slight increase in the category “below lower natural limit” (below 0.3 mIU/L) was found (6.8% in the morning (7–8am) vs. 7.9% in the afternoon (2–3pm)), whereas a substantial decrease for the category “above upper natural limit” (above 4.0 mIU/L) was found decreasing from 14.1% (7–8am) to 7.9% (2–3pm).

Rest was found to have a significant impact on TSH (p = 0.004), calcium (p = 8.9×10^−7^) and sodium (p = 8.7×10^−16^). A significant change was also found for albumin but, as the entire change here occurred within the first 10 minutes of rest, the linear regression solely included patients who rested for 0–10 minutes (p = 2.6×10^−4^; n = 15,544).

**Fig 3:**
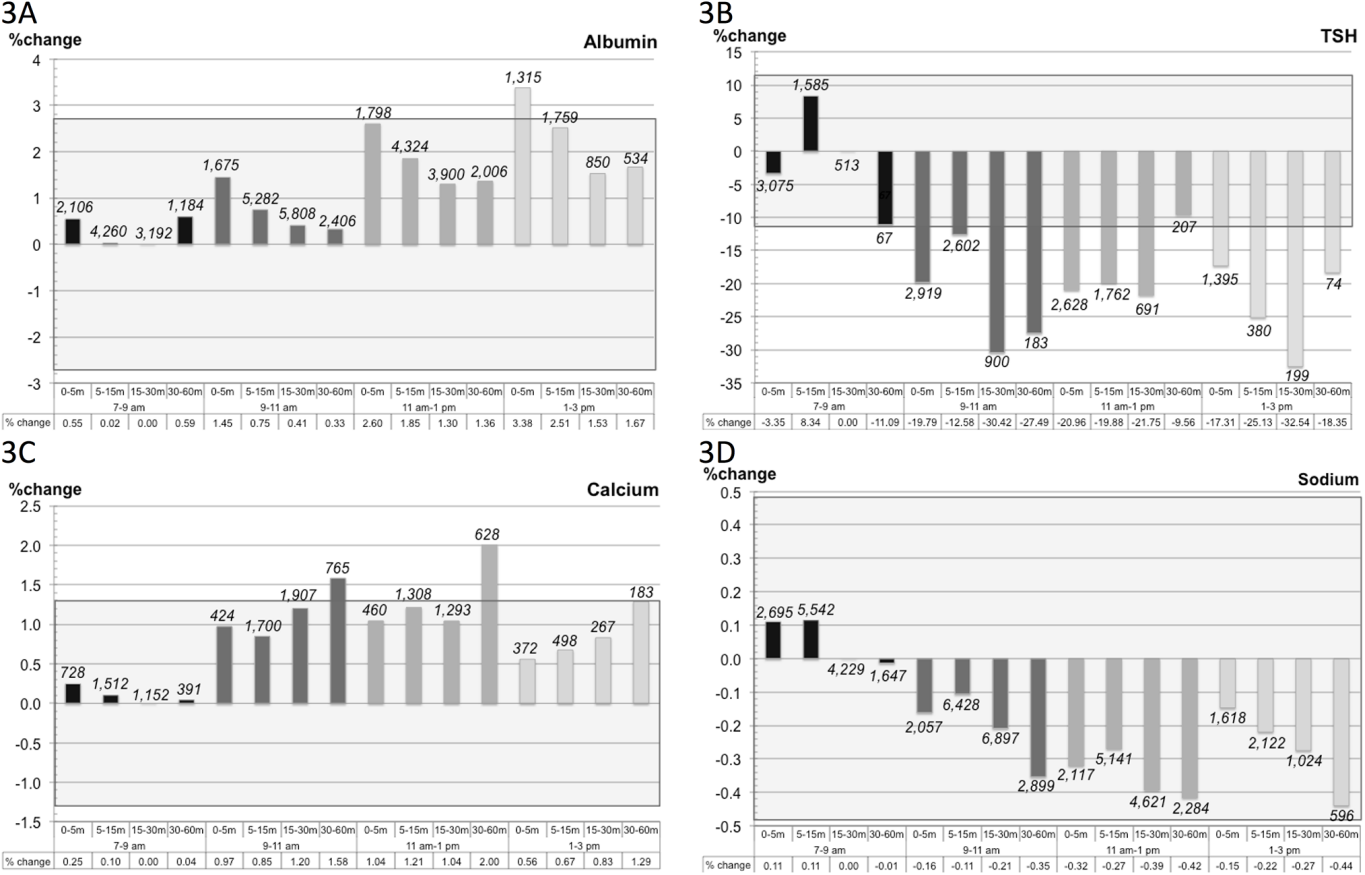
Total impact of resting time and diurnal variation on the biochemical compounds. The figure shows the change in mean value (in %) relative to the mean under the reference conditions (7–9am, resting time 15–30 minutes) for the four components A) albumin, B) thyrotropin, C) calcium and D) sodium. The shadowed background field indicates the limits for the MAB (1/3×SD_pop_). The values above the columns are the number of patients in each interval.

Fig 3 shows the combined effect of resting time and diurnal variation for the four components. The results for albumin and sodium show that only a few patient subgroups failed to fulfil the MAB. All mean values for calcium from 9am–3pm for the interval 30–60 minutes fall outside the limit of the MAB. For TSH the change was noticeable and after 9am all but one patient subgroup failed to fulfil the MAB criteria.

## Discussion

The current study aimed to investigate the influence of resting time and diurnal variation on four common biochemical components obtained from an outpatient blood sampling clinic. We found significant changes for all these analytes (albumin, TSH, total calcium and sodium) in respect to both resting time and diurnal variation. Despite significant results, the mean changes were only moderate and within limits of the MAB for the short resting time intervals (0–5 minutes and 5–15 minutes for all subgroups for calcium and sodium and all but one for albumin (Fig 3). We conclude that our results do not support the current recommendation for patients to rest for a standardised period of at least 15 minutes prior to blood sampling [5,6]. Moreover, when sampling for calcium, a shorter resting time might be desirable as this has to be done within 30 minutes of rest (from 9am–3pm all mean values for the interval 30–60 minutes fall outside the limit for the MAB).

A smaller variation in mean was observed for albumin, sodium and calcium (<3%) relative to TSH (28%) (Fig 1). Serum electrolytes (calcium and sodium) are kept homeostatic under normal conditions e.g. they are regulated [9]. The same applies to albumin, although albumin has a circadian rhythm [10,11]. Under normal conditions the regulation ensures that the components are maintained within narrow limits [9,10] whereas TSH are released in a pulsatile fashion and have a more pronounced circadian rhythm [3]. It would therefore be expected that regulated homeostatic components would be relatively stable, whereas hormones as TSH might vary substantially [9,10,11,12]. This is expected to explain the smaller variation in means observed for albumin, sodium and calcium (<3%) compared to TSH (28%) (Fig 1).

Both albumin and TSH had diurnal variation (Fig 1), which correlates with the results of previous studies [3,11]. For albumin the diurnal variations may be due to varying hydration and metabolism during the day [11]. Our results showed for albumin, a linear increase in the mean value during the day, although the MAB was not exceeded until around 2–3pm (closing time of the outpatient clinic) (Fig 1A). TSH is as mentioned earlier pulsatile secreted and the TSH-releasing hormone from hypothalamus as well as dopaminergic and somatostatinergic mechanisms controls this secretion. According to Behrends *et al*. it is likely to be the dopaminergic tone that plays a major role in the diurnal variation of TSH [13]. For TSH we observed, a considerable decrease in mean value between 7–9am, after which time mean values were stable (Fig 1B). The change in TSH values exceeded the MAB and affected the classification of patients in respect to the reference interval, especially the fraction of patients with values above 4.0 mIU/L. Thus when comparing patients whose blood was sampled during the morning compared to those whose blood samples were taken in the afternoon, more patients were measured at above 4.0 mIU/L in the morning than in the afternoon (14.1% at 7–8am vs. 7.9% at 2–3pm) (Fig 2). A similar but less pronounced shift in classification was observed for individuals with values below 0.3 mIU/L (6.8% in the morning (7–8am) vs. 7.9% in the afternoon (2–3pm)). For calcium and sodium only minor diurnal variations occurred.

The results confirm the known significant diurnal variation for the investigated components. However, a high level of significance does not necessarily imply clinical importance. Therefore, to estimate whether this was the case, we set up a “quality indicator”: For the reference interval to be valid at any given time, the mean change had to be ≤1/3×SD_pop,_ (MAB) [6,7,8].

For albumin the MAB was not exceeded until around 2–3pm (closing time of the outpatient clinic) (Fig 1B). We deduced that, as long as opening hours are not extended, application of the common reference interval is not challenged. However, as the hospital serves both in- and outpatients, it is crucial that reference intervals provided by the laboratory can be used on a 24-hour basis. It may therefore be necessary to reconsider the validity of the reference interval when used on a 24-hour basis. These findings are supported by a previous study by Petersen *et al*. showing that MAB would be exceeded during the interval 2pm–10pm for albumin [11].

As mentioned above, the mean value for TSH decreased from 7–9am, followed by stabilisation (Fig 1B). This correlates well with an earlier study, where a considerable decrease during the morning was reported [3]. From 10am to 3pm all mean values exceeded the limit for the MAB, indicating that there was a clinical impact in relation to the classification of patients (Fig 1B). In fact, when comparing the morning (7–8am) with the afternoon (2–3pm), 6.2% of the patients were misclassified in the afternoon at the higher end of the reference interval (4.0 mIU/L) and 1.1% at the lower end (0.3 mIU/L). This clearly shows the diurnal effect (decrease in the morning followed by stabilisation). By consequence, the blood sampling procedure should either 1) comply strictly with the guidelines (taking the blood samples in the early hours during the morning [3]) or 2) the reference interval should be adjusted to match to the optimal time interval, e.g. 9am to 3pm. Since taking blood samples for only two hours a day is impossible and (possibly more importantly) since there is a smaller diurnal variation during the period 9am–3pm, we prefer the latter solution.

For calcium (Fig 1C) and sodium (Fig 1D), the MAB was never exceeded, and therefore, the diurnal changes are of no clinical importance for these components.

We conclude that the reference interval for albumin should be changed in one of the following ways; either by 1) establishing a floating reference interval that fluctuates continually throughout the day or by 2) dividing the day into e.g. two-hour intervals and establishing reference intervals for each interval or 3) having a 24-hour defined reference interval. For TSH the diurnal variation was found to be pronounced and clinically important in relation to patient classification (Fig 2). A change of procedure is necessary and could be done in the same way as described for albumin.

Fig 3A shows the combined effect of resting time and diurnal variation for albumin. The figure depicts the change in mean value (%) relative to mean under the reference conditions (7–9am, 15–30 minutes’ resting time). The linear increase in the mean value observed for the diurnal variation alone is also apparent here. Moreover, there also seemed to be a pattern in the resting time. This pattern was a decrease in the first 0 to “5–15 minutes” followed by stabilisation. This correlated well with results from the linear regression, where a decrease was found within the first 10 minutes, after which time no significant change occurred.

For TSH, calcium and sodium, the correlation between resting time and the laboratory values was not as clear (% change, Fig 3B–3D). The changes in mean values for TSH exceeded the limit for almost all patient subgroups, mainly due to diurnal variation. For calcium the changes in mean values for two patient subgroups exceeded the limit–both were 30–60 minutes’ resting time subgroups. One could argue that for calcium it seems best to take the sample prior to 30 minutes of resting time.

For sodium all the changes in mean values were inside the limits and therefore of no clinical importance.

One of the strengths of the current study is that it has solid data material based on a very large number of blood samples and a clear set of inclusion and exclusion criteria. For data analysis, patients under the age of 20 (TSH) or 15 years (albumin) were excluded due to age-specific differences in reference intervals. Patients that were also having an ECG were excluded from data analysis as the data did not show whether the ECG was performed before or after the blood sampling. If the ECG was performed prior to blood sampling, this would give a false indication of the resting time. Only patients waiting between 0–60 minutes were included in the data analysis as only a minority of patients waited 60 minutes or more and such a prolonged waiting time increases the probability that they moved around.

Prior to data analysis we compared the age distribution of patients attending our outpatient clinic for blood sampling in the opening hours and found no major differences. Therefore, we assume homogeneity of the population throughout the time interval 7am–3pm.

The study has weaknesses in that 1) the interval 7am–3pm is given by the clinic opening hours (the hospital serves ambulant and admitted patients so it is crucial that reference intervals can be used on a 24-hour basis) and 2) it includes only outpatients. Furthermore, estimating resting time from the waiting time possibly introduces a bias since patients might not all rest all the time while waiting. An ideal study-set-up would have been to do many longitudinal time studies of patients. Therefore it is a limitation to this study that such a procedure would be difficult to carry out because of economical and patient acceptance problems. On the other hand our main focus was on the population level, not on the individual.

In conclusion, this study found no need for resting 15 minutes prior to blood sampling for the examined biochemical components. However, diurnal variation was found to have considerable impact on TSH and, to a minor degree, albumin even within the opening hours 7am–3pm. These variations have to be taken into account to ensure that reference intervals provided by the laboratory can be applied on a 24-hour basis.

## Supporting Information

S1 File Data sets for TSH, albumin, total calcium and sodium. Data sets for the period from November 2011 to June 2014 (during opening hours: 7am–3pm). In-and exclusion criteria: Only patients who waited from 0–60 minutes were included and patients arriving for ECG-measurements were excluded. 1) Albumin, *n = 42*,*399*. Patients under the age of 15 years were excluded. 2) TSH, *n = 20*,*019*. Patients under the age of 20 were excluded. 3) Total calcium, *n = 13*,*588*. 4) Sodium, *n = 51*,*917*.
(XLSX)

## Abbreviations

ECG: Electrocardiogram
MAB: Maximum allowable bias
SD_pop_: Standard deviation (in the population)
TSH: Thyrotropin
WG: Working groups
